# Synergistic prognostic value of coronary distensibility index and fractional flow reserve based cCTA for major adverse cardiac events in patients with Coronary artery disease

**DOI:** 10.1186/s12872-022-02655-0

**Published:** 2022-05-14

**Authors:** Xiao-long Zhu, Zhi-ying Pang, Wei Jiang, Ting-yu Dong

**Affiliations:** 1 Department of Medical Imaging, The First Affiliated Hospital of Hebei North University, No. 12, Changqing Road, Qiaoxi District, Zhangjiakou, 075000 Hebei China; 2 Graduate School of Hebei North University, Hebei, China; 3 The Medical Engineering Office, The First Affiliated Hospital of Hebei North University, Hebei, China

**Keywords:** Coronary artery disease (CAD), Coronary distensibility index (CDI), Fractional flow reserve (FFR), Coronary computed tomographic angiography (cCTA), Major adverse cardiac events (MACEs)

## Abstract

**Background:**

Coronary distensibility index (CDI), as an early predictor of cardiovascular diseases, has the potential to complement coronary computed tomography angiography (cCTA)-derived fractional flow reserve (CT-FFR) for predicting major adverse cardiac events (MACEs). Thus, the prognostic value of CT-FFR combined with CDI for MACEs is worth exploring.

**Methods:**

Patients with a moderate or severe single left anterior descending coronary artery stenosis were included and underwent FFR and CDI analysis based on cCTA, followed up at least 1 year, and recorded MACEs. Multivariate logistic regression analysis was performed to determine independent predictors of MACEs. The area under of receiver operating characteristic (ROC) curve was used to evaluated evaluate the diagnostic performance of CT-FFR, CDI, and a combination of the two.

**Results:**

All the vessel-specific data were from LAD. 150 patients were analysed. 55 (37%) patients experienced MACEs during follow-up. Patients with CT-FFR ≤ 0.8 had higher percentage of MACEs compared with CT-FFR > 0.8 (56.3% vs.7.3%, *p* < 0.05). Patients’ CDI was significantly decreased in MACEs group compared with non-MACEs group (*p* < 0.05). Multivariate analysis revealed that diabetes (*p* = 0.025), triglyceride (*p* = 0.015), CT-FFR ≤ 0.80 (*p* = 0.038), and CDI (*p* < 0.001) are independent predictors of MACEs. According to ROC curve analysis, CT-FFR combined CDI showed incremental diagnostic performance over CT-FFR alone for prediction of MACEs (AUC = 0.831 vs. 0.656, *p* = 0.0002).

**Conclusion:**

Our study provides initial evidence that combining CDI with CT-FFR shows incremental discriminatory power for MACEs over CT-FFR alone, independent of clinical risk factors. Diabetes and triglyceride are also associated with MACEs.

**Supplementary Information:**

The online version contains supplementary material available at 10.1186/s12872-022-02655-0.

## Introduction

Cardiovascular diseases (CVD) cause approximately one-third of deaths in worldwide. Coronary artery disease (CAD), also referred to atherosclerotic cardiovascular disease (ACD). It manifests clinically as myocardial infarction and ischemic cardiomyopathy, and is the commonest type of CVD [1]. Currently, an increasing number of individuals with CAD, and the increasing incidence of ACD is expected to continue [2]. Thus, early detection of CAD is critical to avoid further increases in the risk. Moreover, accurate and effective methods for CAD detection to enhance the accuracy of heart disease diagnosis also become the inevitable trend of modern medical development.

Invasive coronary angiography (ICA) is an established gold standard technique for the diagnosis and treatment of CAD. However, it cannot reveal the hemodynamic significance, and may lead to various complications due to its invasiveness [3, 4]. Besides, currently, the diagnosis methods for CAD also include coronary computed tomography angiography (cCTA), coronary magnetic resonance angiography (CMRA), intravascular ultrasound (IVUS), and optical coherence tomography (OCT) [5–7]. Terribly, these methods only provide anatomic assessment of stenosis and are a poor predictor of the hemodynamic significance of coronary artery stenosis with a tendency to overestimate stenosis severity, which is critical for determining whether a lesion requires revascularization [8–10]. Overestimate stenosis severity increased the risk of percutaneous coronary intervention (PCI) for patients with coronary artery stenosis, and increased the financial burden on patients.

Excitingly, fractional flow reserve (FFR) has been considered to be a gold standard for assessing the hemodynamic significance of CAD. Moreover, FFR is also associated with major adverse cardiac events (MACEs), and CT-FFR ≤ 0.8 is a better predictor of clinical outcomes. Several studies of cCTA based FFR (CT-FFR) have demonstrated that CT-FFR has a high diagnostic value for stenosis assessment, and are superior to alone cCTA in predicting myocardial ischemia, particularly in terms of improved specificity and positive predictive value [11–14]. In addition, a deep learning (DL) algorithm based CT-FFR further improves the diagnostic accuracy for hemodynamic changes of stenosis by its self-learning ability [11]. However, the limitations of CT-FFR is it is not able to accurately reflect the coronary artery elasticity (CAE), which affected the accuracy of CT-FFR for predicting ischemic cardiomyopathy, and remains prone to inaccurate calculation of FFR [15]. To overcome these pitfalls, the combination of coronary distensibility index (CDI) is necessary.

CDI, a measure index of CAE, can serve as a predictive marker for the risk of coronary heart disease in human beings. A previous study demonstrated that CDI is inversely related to the severity of CAD [16]. Additionally, several studies indicated that an early sign of atherosclerotic change in arterial distensibility reduction [17, 18]. Thus, CT-FFR combined with CDI may early detect the functional changes in CAD [15]; thus, we speculated that CDI may also improve the accuracy of CT-FFR method to predict MACEs. However, the prognostic value of CT-FFR combined with CDI based on cCTA for the prediction of MACEs is not known and requires further investigation.

Therefore, the present study aimed to compare the prognostic value of CT-FFR, CDI, and CT-FFR with CDI in predicting MACEs. Meanwhile, evaluate the diagnosis capabilities of CT-FFR combined with CDI based on cCTA for MACEs.

## Method

### Patients

In this retrospective study, we pooled clinical data on 150 patients from a single center between January 2017 and August 2020. Patients had received oral medication and were diagnosed with a moderate or severe single left anterior descending coronary artery stenosis (stenosis degree 50%-90%) by coronary CTA examination. Meanwhile, we measured the fractional flow reserve (FFR) and coronary distensibility index (CDI) of the 150 patients based on coronary computed tomographic angiography (cCTA).

The study had been approved by the Ethics Committee of the First Affiliated Hospital of Hebei North University (Ethical approval number: W2017069) and written informed consent had been obtained from all research patients before enrollment.

Eligible patients were moderate or severe single left anterior descending coronary artery stenosis, and the right coronary artery (RCA) or the left circumflex coronary artery (LCX) stenosis was mild and without high-risk plaque. Other eligibility criteria were patients for whom the cCTA was adequate quality for CT-FFR and CDI analysis, and patients with complete hospital records. In addition, eligible patients were received oral medication. However, patients were not considered for study inclusion if they had received percutaneous coronary intervention (PCI) or coronary artery bypass graft (CABG). Patients who were suspected of acute coronary syndrome, cardiac arrhythmia, multiple coronary artery lesions (moderate/severe) were ineligible for this study. In addition, patients whose cCTA imaging cannot perform assessments of CT-FFR and CAE were not allowed to include.

### cCTA acquisition and analysis

Standard acquisition and analysis protocols were based on available international guidelines [19, 20]. Metoprolol was administered orally 1–2 h before CCTA to control a heart rate of < 70 beats/min. The anteroposterior and lateral position of chest image acquisition was performed using Aquilion 320-MDCT after administering 60–80 mL of nonionic contrast iodixanol with a concentration of 320 mg/mL and 20 mL of normal saline intravenous at a rate of 4–5 mL/s. Imaging was started below the carina of the trachea and continued to 1 inch below the bottom of the diaphragmatic surface of the heart. Data acquisition was performed for 7–10 s with 120-kV tube voltage, 440- mA tube current, and slice thickness of 0.5 mm. The raw data files were transferred to the Vitrea workstation and reconstructed using thin slices 0.5 mm and medium smooth reconstruction filters in 75% phases for the generation of 3D CTA models. Then, 3D CTA images were reconstructed by multi-planar reconstructions (MPR), maximum intensity projection (MIP), curved planar reformations (CPR), and volume rendering technique (VRT) using the Vitrea workstation.

### CDI acquisition and analysis

**Fig. 1:**
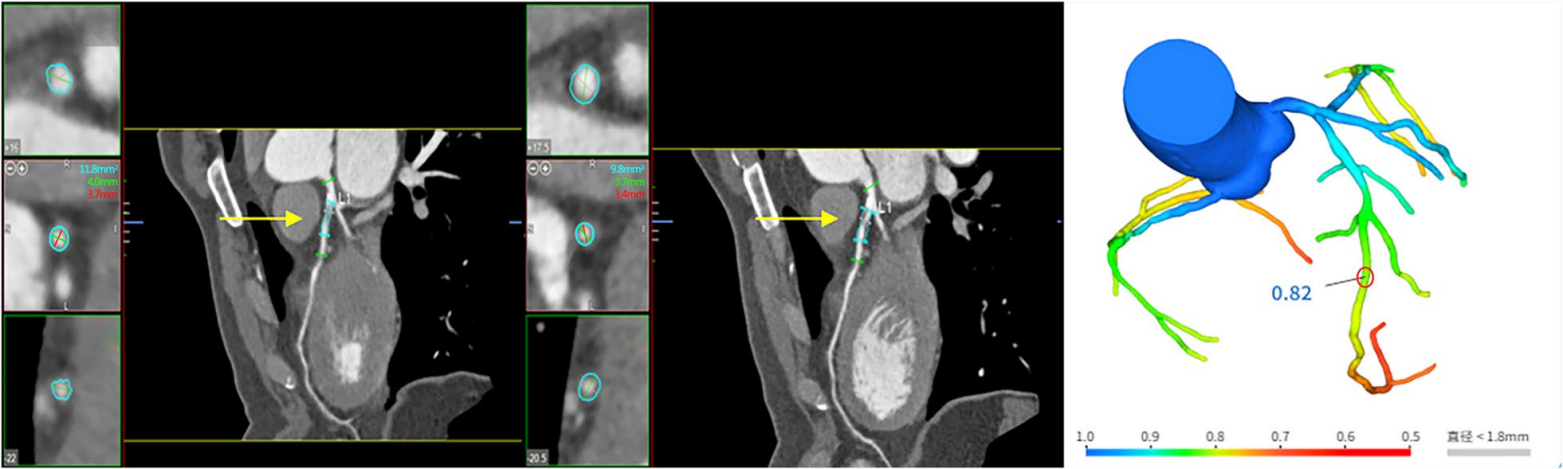
A case example of coronary stenosis on cCTA-based CDI and CT-FFR analysis. A 53-year-old man without MACEs who underwent CDI and CT-FFR analysis based on cCTA. **A** CDI analysis based on cCTA demonstrated that the lumen area was 11.8 mm^2^ at the early diastolic (40%) of the left anterior descending (LAD) artery 5 mm distal to the narrowest of the left main coronary artery. **B** CDI analysis based on cCTA demonstrated that the lumen area was 9.8 mm^2^ at the mid diastolic (75%) of the LAD artery 5 mm distal to the narrowest of the left main coronary artery. **C** shows no hemodynamically significant LAD lesion with a CT-FFR value of 0.82. The green data indicates the maximum diameter and red data indicates the minimum diameter. The red circle indicates that the measured position is the left anterior descending branch located distally 2 cm to the stenosis. MACEs, major adverse cardiac events; cCTA, coronary computed tomography angiography; CDI, coronary distensibility index; CT-FFR, cCTA-derived fractional flow reserve

The cCTA data were reconstructed from 30 to 75% of the R-R interval with 5% intervals increments. Early diastolic and mid diastolic (MD) cross-section area (CSA) of the left anterior descending (LAD) artery were measured 5 mm distal to the narrowest of the left main coronary artery by two independent experienced investigators (Fig. 1A, B). Repeat the measurement three times. The Smax and Smin in 30% to 75% of the R-R interval was obtained using the vascular analysis software. CDI was the change of lumen cross-sectional area of coronary artery under unit pressure and calculated using the following equation:

$${\text{CDI}} = \frac{{{\text{S}}_{{{\text{max}}}} - {\text{ S}}_{{{\text{min}}}} }}{{{\text{S}}_{{{\text{min}}}} \times \left( {{\text{P}}_{{\text{s}}} - {\text{P}}_{{\text{d}}} } \right)}} \times 10^{ - 3}$$

S_max_ is defined as the maximum cross-sectional area of the regions of interest (ROI) in the vascular lumen, S_min_ as the minimal cross-sectional area of the regions of interest (ROI) in vascular lumen, P_s_ represents systolic pressure, and P_d_ represent diastolic pressure.

### Deep learning-based CT-FFR

The CT- FFR was analyzed using a deep learning-based approach as previously described [21]. The cCTA image data in DICOM (Digital Imaging and Communications in Medicine) format were transferred to the platform in Keya medical technology Inc. (Shenzhen, China). The three-dimensional coronary artery model and its centerlines were first extracted and then creation of a patient-specific colored-coronary tree (different colors represent different thresholds of CT-FFR values), the DEEPVESSEL-FFR value along the coronary arterial tree was calculated by the deep learning algorithm. The CT-FFR was measured by trained analysts who were blinded to other clinical information, and the measurement location was 2 cm at the distal end of the patient's vascular plaque (Fig. 1C). CT-FFR value 0.80 was considered as hemodynamically significant.

### Follow up and study endpoints

All included patients underwent followed up for at least 1 year, and the longest follow-up was 4.67 years. The occurrence of MACEs was recorded every 3 months. The primary endpoint of this present study is MACEs, and patients were divided into MACEs group and non-MACEs group. MACEs was defined as cardiac death, non-fatal myocardial infarction, rehospitalization due to heart failure or aggravated angina symptom, or revascularization (percutaneous coronary intervention or coronary artery bypass grafting).

### Assessment

The diagnostic performance of CDI, CT-FFR, and CDI combined CT-FFR for MACEs were assessed in terms of the area under the ROC curve (AUC), sensitivity, and specificity. AUC value represents the predictive probability of the CDI, CT-FFR, and a combination of the two (AUC = 0.5, none; AUC: 0.5–0.7, poor; AUC: 0.7–0.9, reasonable; AUC > 0.9, high). Sensitivity is defined as the proportion of true positives that were correctly predicted by the model as having the diagnosis, while specificity is the proportion of true negatives that were correctly predicted by the model. A higher AUC corresponding to a higher overall diagnostic accuracy [22]. AUC were compared using the method of DeLong et al. [23].

### Statistical analysis

Descriptive statistics were provided using medians (ranges) and means (standard deviations) for continuous variables and frequency (proportions) for categorical variables. Analysis of nominal variables was performed using the chi-square test or Fisher exact test for between-group comparisons. Comparison of continuous variables between two groups was analyzed using t-test. Logistic regression analysis was performed to determine independent predictors of MACEs. Diagnostic performance of the CDI, CT-FFR, and CT-FFR combined CDI was assessed by constructing a receiver operating characteristic (ROC) curve and evaluated by calculating the area under curve (AUC), and reported with 95% confidence intervals (CIs). All statistical tests are two-sided, *p* value of < 0.05 was considered statistically significant. All statistical analyses were conducted using SPSS software version 22.0.

## Results

### Patient characteristics

The study population consisted of 150 patients with stenosis of the left anterior descending coronary artery (stenosis degree 50–90%), and received oral medication. The baseline demographics and clinical characteristics of the overall study population and the MACEs versus non-MACEs groups are given in Table 1. The mean age of the 150 study population (80.7% men) was 63.3 years ± 9.8, and the mean follow-up period was 18.2 months (IQR 11.2–25.8). Of these 150 patients, 55 (37%) experienced MACEs in their follow-up period while 95 patients (63%) with no MACEs. Of the MACEs patients, 3 patients (5.5%) experienced sudden cardiac death (SCD), 36 patients (65.5%) received the percutaneous coronary intervention (PCI) or coronary artery bypass grafting (CABG), 13 patients (23.6%) were hospitalized again for worsening of the symptoms, including angina (11) and cardiac failure (2). Additionally, of all 150 patients, 21(14%) patients have mild LCX stenosis, while 40 (26.7%) patients have mild RCA stenosis. The high-risk plaque characteristics in LAD mainly including positive remodeling (36%), spotty calcification (28.7%), napkin-ring sign (20%), and low attenuation plaque (16%) (Additional file: Table S1 [Information about the disease status of vessels between patients with and without MACEs]).

**Table 1:** Overview of the patient demographics

	MACEs group (*n* = 55)	Non-MACEs group (*n* = 95)	*P* value	Total (n = 150)
Age	62.5 ± 9.6	63.8 ± 9.3	0.840	63.3 ± 9.8
Female (%)	49 (89.1)	72 (75.8)	0.513	121 (80.7)
BMI (kg/m^2^)	25.0 ± 3.0	24.9 ± 2.8	0.920	25.1 ± 2.9
**Cardiovascular risk factors**				
Hypertension (%)	45 (81.8)	79 (83.2)	0.513	124 (82.7)
Diabetes (%)	39 (70.9)	63 (66.3)	0.048	102 (68)
History of smoking (%)	32 (58.2)	41 (43.2)	0.344	73 (48.7)
HCY(umol/L)	15.8 ± 9.8	15.3 ± 8.9	0.569	19.1 ± 10.3
HGB (g/L)	148.1 ± 19.7	149.6 ± 15.1	0.863	148.57 ± 18.4
Scr (umol/L)	69.1 ± 11.4	71.0 ± 12.1	0.915	70.9 ± 11.6
TG (mmol/L)	1.47 ± 0.44	1.24 ± 0.28	0.001	1.36 ± 0.36
TCH (mmol/L)	4.7 ± 1.0	4.6 ± 1.1		4.6 ± 0.9
HDL-C (mmol/L)	1.1 ± 0.2	1.2 ± 0.3	0.468	1.1 ± 0.3
LDL-C (mmol/L)	3.1 ± 0.9	3.0 ± 1.0	0.203	2.9 ± 1.0
ApoA1 (g/L)	1.1 ± 0.2	1.2 ± 0.1	0.055	1.15 ± 0.2
ApoB (g/L)	1.0 ± 0.2	1.0 ± 0.3	1.339	0.96 ± 0.3
CT-FFR			0.038	
CT-FFR ≤ 0.8	46 (83.6)	58 (61.1)		104 (69.3)
CT-FFR > 0.8	9 (16.4)	37 (38.9)		46 (30.7)
CDI (× 10^–3^/mmHg)	3.5 ± 0.6	4.0 ± 0.7	0.003	3.8 ± 0.7
**MACEs (n = 55)**				
Death				3 (5.5)
Angina				11 (20)
Cardiac failure				2 (3.6)
PCI				34 (61.8)
CABG				5 (9)

Data were expressed as mean (standard deviation) or n (%).

*BMI* body mass index, *HDL-C* high-density lipoprotein-cholesterol, *HCY* Homocysteine, *HGB* hemoglobin, *Scr* serum creatinine, *TG* triglyceride, *TCH* total cholesterol, *LDL-C* low-density lipoprotein- cholesterol, *ApoA1* apolipoprotein A1, *ApoB* apolipoprotein B, *CT-FFR* computed tomography-fractional flow reserve, *CDI* coronary distensibility index, *PCI* percutaneous coronary intervention, *CABG* coronary artery bypass grafting

### Correlation of cardiovascular risk factors, CT-FFR and CDI with MACEs

The percentage of patients with a history of diabetes was higher in the MACEs group compared to the non-MACEs group (70.9% vs. 66.3%, *p* < 0.05). In addition, the triglyceride levels has a higher in MACEs group versus the non-MACEs group (*p* < 0.05). Thus, patients with dyslipidaemia and diabetes mellitus were more likely to experienced MACEs during follow-up. Patients with CT-FFR ≤ 0.8 were more likely to have higher percentage of MACEs compared with CT-FFR > 0.8 (69.3% vs. 30.7%, *p* < 0.05). The MACEs group had 46 patients (83.6%) with CT-FFR values ≤ 0.8 indicating functional ischemia, and 9 patients (16.4%) with CT FFR > 0.8 showed no significant change in hemodynamic. Comparison of patients in non-MACEs group, the CDI was significantly decreased in patients of MACEs group (*p* < 0.05) (Table 1).

Multivariate logistic regression models were used to analyze the correlation of cardiovascular risk factors (diabetes and triglyceride), CT-FFR of ≤ 0.8, and CDI with MACEs (Table 2). In the univariate analysis, diabetes (OR: 1.03, 95% CI: 1.00–1.06, *p* = 0.043) and triglyceride (OR: 3.01, 95% CI: 1.28–6.73, *p* = 0.009) were related to MACEs. CT-FFR ≤ 0.80 (OR: 3.66, 95% CI; 1.81–7.42, *p* < 0.001) and CDI (OR: 0.16, 95% CI; 0.082–0.031, *p* < 0.001) were significant predictors of MACEs. Multivariate analysis showed that the diabetes (OR: 1.50, 95% CI: 1.01–1.10, *p* = 0.025) and triglyceride (OR: 5.82, 95% CI: 1.80–21.44, *p* = 0.015) were the most closely independent risk factors for MACEs, which were significantly associated with the occurrence of MACEs. CT-FFR ≤ 0.80 (OR: 2.33, 95% CI: 1.05–5.17, *p* = 0.038) and CDI (OR: 0.19, 95% CI: 0.096–0.38, *p* < 0.001) are the independent predictors of MACEs, with a significant in the prognostic value. A case example of coronary stenosis on cCTA, cCTA-based CDI and FFR analysis is illustrated in Fig. 1.

**Table 2:** Analysis of the correlation of cardiovascular risk factors, CT-FFR and CDI with MACEs

Variable	Univariate analysis		Multivariate analysis	
	Odds ratio (95% CI)	*P* value	Odds ratio (95% CI)	*P* value
Diabetes	1.03 (1.00,1.06)	0.043	1.50 (1.01,1.10)	0.025
TG (mmol/L)	3.01 (1.28, 6.73)	0.009	5.82 (1.80,21.44)	0.015
CT-FFR ≤ 0.80	3.66 (1.81, 7.42)	< 0.001	2.33 (1.05–5.17)	0.038
CDI	0.16 (0.082,0.31)	< 0.001	0.19 (0.096,0.38)	< 0.001
Age	0.79 (1.03,1.97)	0.103		
Gender	1.36 (1.43,2.90)	0.612		
BMI	2.51 (1.13–3.38)	1.514		
Hypertension	1.32 (0.87,1.69)	0.067		
History of smoking	2.77 (1.51–4.48)	0.235		

### The prognostic value of CDI, CT-FFR and CDI combined CT-FFR for MACEs

**Fig. 2:**
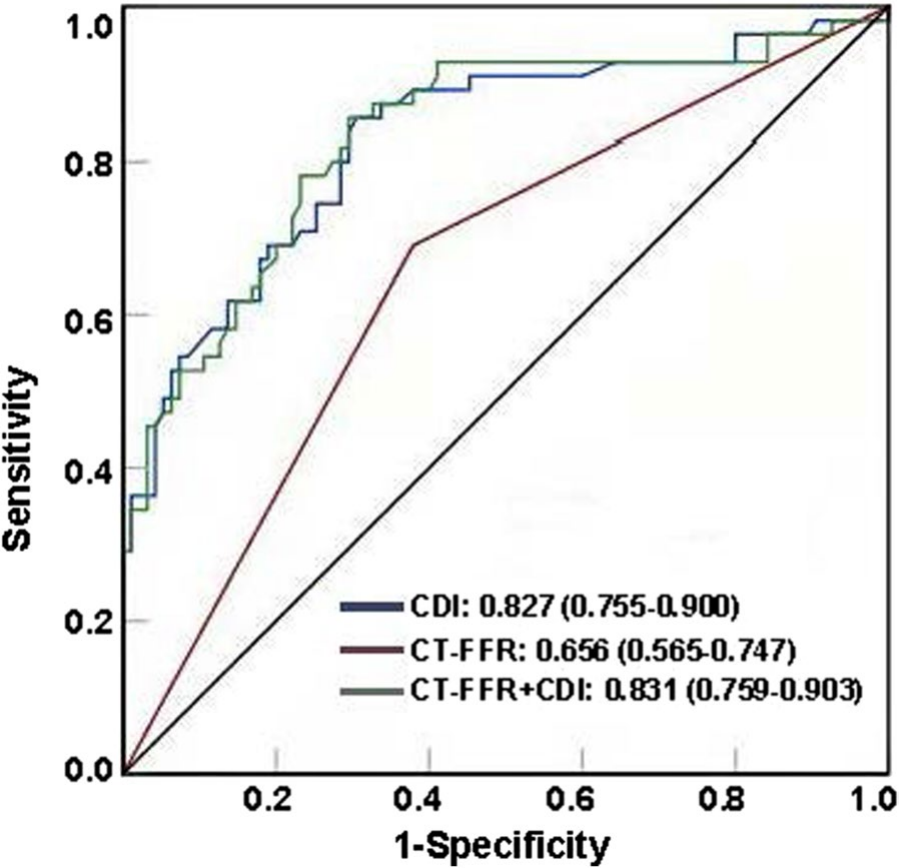
ROC analysis of CDI, CT-FFR and CT-FFR combined with CDI for prediction of MACEs. The CDI alone (blue line) for prediction of MACEs with an area under the curve (AUC) of 0.827, the CT-FFR alone (red line) with an AUC of 0.66, and CT-FFR combined with CDI (green line) resulting in the highest predictive value with an AUC of 0.831

ROC curves was performed to evaluate the predictive value of CDI, CT-FFR and CDI combined CT-FFR for MACEs. In the ROC analysis for MACEs, the area under the curve (AUC) value of combined CDI and CT-FFR approach was higher than CT-FFR alone (0.831 vs.0.656, 95% CI: 0.102–0.248, *p* < 0.0001), similarly, the AUC value of CDI alone was also higher than CT-FFR alone (0.827 vs.0.656, 95% CI: 0.813–0.261, *p* = 0.0002 < 0.05). However, there is no significant difference between the AUC value of the combining CDI with CT-FFR approach and the CDI alone approach (*p* = 0.7 > 0.05). The highest sensitivity (90.2%, 95% CI: 81.3–98.7) and specificity (91.5%, 95% CI: 83.1–97.4) for MACEs prediction was acquired with CDI combined with CT-FFR. In addition, the sensitivity and specificity of CDI alone was 88.5% and 89.3% (95% CI: 86.4–89.2/84.6–91.4), and CT-FFR alone was 45.7% and 85.3% (95% CI: 31.2–60.9/79.6–91.1). Overall, CDI combined CT-FFR showed favorable diagnostic performance and incremental discriminatory power over CT-FFR alone for the prediction of MACEs (Fig. 2).

## Discussion

The present study evaluated the prognostic value of CT-FFR, CDI, and CT-FFR combined with CDI for MACEs. The study results demonstrated that CT-FFR and CDI measured based on cCTA, and a combination of the two, all carry predictive values to identify MACEs. Most importantly, the combined approach of CDI together with CT-FFR determination yielded incremental predictive value for MACEs. Moreover, diabetes and triglyceride were significantly associated with the occurrence of MACEs in the present study.

Multiple studies confirmed CT-FFR is a better predictor of MACEs in patients with CAD, especially for patients with intermediate-range or severe-range coronary stenosis (≥ 50%). Moreover, patients with CT-FFR ≤ 0.80 had a higher risk of MACEs than patients with CT-FFR > 0.80 [5, 11, 24, 25], which was consistent with our study. We suspected that CT-FFR can provide high sensitivity and specificity to identify myocardial ischemia, CT-FFR ≤ 0.80 can indicate functional ischemia, while myocardial ischemia was associated with a trend towards an increase in MACEs risk. A recent study also suggested that the annualized rate of MACEs was higher for patients with myocardial ischemia, which was independent predictor of MACEs [26]. Of note, previous studies demonstrated that in patients with intermediate-range coronary stenosis, CT-FFR > 0.80 can be effective in differentiating patients who do not require further diagnostic testing or intervention, and none of these patients experienced MACEs within 3 years [27, 28]. However, the present study results showed that in 35 patients of intermediate-range or severe-range coronary stenosis with MACEs, 16.4% patients with CT-FFR > 0.8. A previous study showed that FFR_CT_ is vessel-level hemodynamic parameters, and the estimation of FFR is its dependent on achieving maximal hyperemia. Failure to achieve peak hyperemia may result in not achieving minimal constant microvascular resistance, leading to underestimation of pressure drop and overestimation of FFR across a stenosis [29, 30]. The local pressure drop is the pressure of each lesion, which may indicate a focal lesion without maximal hyperemia, and may provide guidance for the distinguishing local and diffuse forms of coronary artery disease, especially for the patient population with non-ischemic FFR value [31]. Additionally, EMERALD study highlighted the prognostic importance for the acute coronary syndrome (ACS) of the definition of focal pressure [32]. Therefore, we suspected that the results (16.4% patients with CT-FFR > 0.8 experienced MACEs) may be associated with a focal lesion. However, it is necessary to investigate with large sample sizes in future study. In addition, we also suspected that the diagnostic accuracy of CT-FFR can be affected by the uncontrollable factor (such as image quality, lesions). A systematic review showed that the diagnostic accuracy was only 46.1% for CT-FFR values between 0.70 and 0.80, which was determined as a grey zone of CT-FFR [33]. A substudy of the NXT trial indicated that lesions with large or diffuse calcification may affect the coronary tree extracted and the resultant calculation of CT-FFR [34]. Thus, poor diagnostic accuracy of CT-FFR for MACEs prediction can be affected by the image quality and a grey zone. This also supported our findings. Furthermore, CT-FFR is not able to accurately reflect the coronary artery elasticity (CAE), which also affected the accuracy of CT-FFR for predicting ischemic cardiomyopathy [16]. Hence, it is necessary to combine clinical and other imaging risk predictors to assess the risk of MACEs and guide clinical decisions, rather than relying solely on a single parameter.

The important finding of the present study demonstrated that the diagnostic performance of CT-FFR for MACEs was improved by combining it with CDI, which has not been previously reported. To our knowledge, CDI can assess underlying vascular dysfunction, and also monitor potential changes in vascular function in response to the occurrence of adverse cardiovascular events. Previously study showed that the CDI measured using cCTA was found to have a strong correlation with quantitative coronary angiography, as well as an inverse relationship between distensibility and the severity of CAD [33]. Thus, the strong correlation between CTA and CDI suggests CDI may be a potentially useful noninvasive addition to the current CTA cardiovascular assessment. Additionally, Haluska et al. indicated that the poor prognosis in patients with various degrees of cardiovascular risk is independently associated with impaired arterial distensibility, and arterial distensibility may identify patients at risk of future MACEs [35]. Therefore, the excellent correlations between arterial distensibility and CAD also support the superior incremental value of CDI for predicting MACEs. This also supports our outcomes. However, the predictive value of CDI alone to MACEs was also with a relatively high AUC of 0.827. Similar to our finding, previous studies also showed that the CDI from the cCTA have a favourable diagnostic performance for the obstructive CAD lesions (AUC = 0.81) and endothelial-dependent microvascular dysfunction (AUC = 0.88) [16, 36]. Notably, there is no significant difference between the AUC value of the combining CDI with CT-FFR approach and the CDI alone approach. Thus, it is necessary to expand the sample size and types of diseased vessels to analyze whether the diagnostic performance of combining CDI with CT-FFR for MACEs is superior to CDI alone. In addition, we also found that diabetes and triglyceride were independent risk factors for MACEs and significantly associated with the occurrence of MACEs. Several studies reported that triglyceride levels and diabetes are independently associated with higher risk of CAD [37–39]. Another study indicated that the triglyceride-glucose index (TyG index) may be an independently useful marker for predicting future MACEs in patients with diabetes and acute coronary syndrome (ACS) [40]. These all supported our findings. Therefore, routinely introducing the CDI into clinical diagnostic models could more effectively and accurately predict the occurrence of MACEs, thus enabling a more targeted treatment or prevention. Meanwhile, triglyceride levels and diabetes is also a potential predictor for MACE in patients with CAD.

Despite the above promising findings, the present study has several limitations. First, as this was a single-center retrospective study with shorter follow-up period, there may be selection bias for the patient population included in this study, and thus influence clinical outcomes. Hence, future prospective studies with longer follow-up period and larger sample size are required to verify the additional value of CDI. Moreover, the CAD patients in this study only with single-vessel disease, and without plaque characteristic analysis. Thus, it is necessary to increase the analysis for sample size of multi-vessel CAD and analysis for plaque characteristics.

## Conclusion

In summary, the current data showed that CDI and CT-FFR was independent predictors of MACEs. Adding the CDI to the CT-FFR analysis has an incremental prognostic value for the prediction of MACEs. In addition, we found that triglyceride levels and diabetes are two independent risk factors for MACEs, thus incorporating diabetes and high triglyceride levels for MACEs prediction might be beneficial in identifying higher risk patients.

## Supplementary Information

**Additional file 1**. Information about the disease status of vessels between patients with and without MACEs.

## Data Availability

All data generated or analysed during this study are included in this published article.
