# Supplementary material for: Synergistic prognostic value of coronary distensibility index and fractional flow reserve based cCTA for major adverse cardiac events in patients with Coronary artery disease
Source: BMC Cardiovasc Disord. 2022 May 14;22:220. doi: 10.1186/s12872-022-02655-0 (PMC9107240; doi:10.1186/s12872-022-02655-0)
Supplement: Supplementary file 1 — Additional file 1. Information about the disease status of vessels between patients with and without MACEs. [file 12872_2022_2655_MOESM1_ESM.docx]

**Supplementary table 1 Information about the disease status of vessels between patients with and without MACEs**

|  | MACEs Group (*n*=55) | Non-MACEs group (n=95) | Total (n=150) | *P* value |
| --- | --- | --- | --- | --- |
| Degree of stenosis in the LAD |  |  |  | <0.001 |
| Moderate, n (%) | 9 (16.4) | 72 (75.8) | 81 (54.0) |  |
| Severe, n (%) | 46 (83.6) | 23 (24.2) | 69 (46.0) |  |
| Distribution and degree of stenosis of other vessels |  |  |  | 0.957 |
| Mild LCX stenosis, n (%) | 8 (14.5) | 13(13.7) | 21 (14.0) |  |
| Mild RCA stenosis, n (%) | 19 (34.6) | 21(22.1) | 40 (26.7) |  |
| Plaque characteristics in LAD |  |  |  | 0.922 |
| Noncalcified plaques, n (%) | 32 (58.2) | 62 (65.3) | 94 (62.7) |  |
| Mixed plaques, n (%) | 26 (27.4) | 34 (35.8) | 60 (40.0) |  |
| Calcified plaque, n (%) | 6 (10.9) | 7 (7.4) | 13 (8.7) |  |
| High risk plaque characteristics in LAD n (%) |  |  |  | 0.948 |
| Positive remodeling | 21 (38.2) | 33 (34.7) | 54 (36.0) |  |
| Napkin-ring sign | 13 (23.6) | 17 (17.9) | 30 (20.0) |  |
| Spotty calcification | 16 (29.1) | 27 (28.4) | 43 (28.7) |  |
| Low attenuation plaque | 9 (16.4) | 15 (15.8) | 24 (16.0) |  |

LAD, left anterior descending artery; LCX, left circumflex coronary artery; RCA, right coronary artery; MACE, major adverse cardiac events.
